# Dietary Patterns and Lifestyle Factors as Determinants of Body Mass Index and Body Composition in Individuals with Down Syndrome—A Study Across Three Clinical Sites

**DOI:** 10.3390/nu18050779

**Published:** 2026-02-27

**Authors:** Maria Gomis-González, Anna Boronat, Klaus Langohr, Leda A. Bianchi, Jasmine Wells, Miren Tamayo-Elizalde, Inés Ben Smida, Laude De Verdun, Li F. Chan, Anne Hiance-Delahaye, André Strydom, Rafael de la Torre, GO-DS21 Consortium

**Affiliations:** 1 Integrative Pharmacology and Systems Neurosciences Research Group, Neurosciences Research Program, Hospital del Mar Research Institute, 08003 Barcelona, Spain; mgomis@researchmar.net (M.G.-G.); aboronat@researchmar.net (A.B.); klangohr@researchmar.net (K.L.); 2 Department of Statistics and Operations Research, Universitat Politècnica de Catalunya, 08028 Barcelona, Spain; 3 Department of Forensic and Neurodevelopmental Sciences, Institute of Psychiatry, Psychology & Neuroscience, King’s College London, London SE5 8AB, UK; leda.bianchi@kcl.ac.uk (L.A.B.); jasmine.wells@kcl.ac.uk (J.W.); miren.tamayo_elizalde@kcl.ac.uk (M.T.-E.); andrestrydom@kcl-ac.uk (A.S.); 4 South London and the Maudsley Foundation NHS Trust, London SE5 8AZ, UK; 5 Institut Jérôme Lejeune, 75015 Paris, France; ines.bensmida@institutlejeune.org (I.B.S.); laude.deverdun@institutlejeune.org (L.D.V.); anne.hiance-delahaye@institutlejeune.org (A.H.-D.); 6 Centre for Endocrinology, William Harvey Research Institute, Faculty of Medicine and Dentistry, Queen Mary University of London, Charterhouse Square, London EC1M 6BQ, UK; l.chan@qmul.ac.uk; 7 Department of Medicine and Life Sciences (MELIS), Universitat Pompeu Fabra, 08003 Barcelona, Spain; 8 Centro de Investigación Biomédica en Red de Fisiopatología de la Obesidad y la Nutrición, Instituto de Salud Carlos III, 28029 Madrid, Spain

**Keywords:** Down syndrome, body mass index, overweight, obesity, diet quality, nutrient intake, physical activity

## Abstract

**Background/Objectives:** It is often reported in the literature that the prevalence of obesity is high in individuals with Down Syndrome (DS). This study aims to assess how lifestyle factors—diet quality, nutrient intake or physical activity—contribute to weight gain. **Methods:** 230 males/females with DS, aged 12–45 years, were recruited across three geographically independent sites. A total of 185 participants were considered for this analysis and classified into normal-weight, overweight, and obese categories. Diet quality and nutrient intake were calculated using country-specific FFQs. Physical activity was assessed with the Minnesota Leisure Time Activity Questionnaire. Body composition measures were obtained with a bioimpedance scale. **Results:** The study corroborates a higher prevalence (%) of overweight/obesity in our DS cohort compared to the general population. Higher BMIs were significantly correlated with older age (*p* < 0.001), lower physical activity (*p* < 0.05), higher parental BMIs (*p* < 0.001, mother’s BMI; *p* < 0.05, father’s BMI), and increased adiposity indicators. Excess body weight showed an inverse association only with protein intake (*p* < 0.001). No significant differences emerged in total caloric or other macronutrients intake across BMI categories. However, notable differences in dietary patterns were observed among the three countries, reflecting cultural influences. A smaller exploratory sub-study suggested a potential relationship between higher IQ scores and better diet quality (*p* < 0.05). **Conclusions:** These findings provide new insights into contributors to overweight/obesity in DS people, indicating an influence of age, physical activity, familial factors, and body composition. Higher protein intake and culturally adapted lifestyle interventions may contribute to improving weight-related outcomes.

## 1. Introduction

Individuals with Down syndrome (DS), the most common genetic cause of intellectual disability, and affecting approximately 1 in every 700–800 live births [1], are at a substantially higher risk of developing various health conditions, including dementia, hypothyroidism, and epilepsy, with reported incidence rate ratios indicating approximately a 95-fold higher risk of dementia, a 10-fold higher risk of hypothyroidism, and a 10-fold higher risk of epilepsy compared with the general population [2].

This population also has an elevated incidence of overweight and obesity, which is a prevalent health concern among these individuals [3,4]. According to the World Health Organization (WHO), an excess of body weight is a distinct risk factor for numerous chronic diseases, such as cardiovascular disease, diabetes, musculoskeletal disorders, and certain cancers.

Due to the numerous medical conditions associated with DS, it may be difficult to establish a direct link between obesity and specific health problems in individuals with this condition. Nonetheless, numerous studies have demonstrated a correlation between weight status in DS and a higher incidence of certain health complications, such as obstructive sleep apnea, metabolic alterations, orthopedic and biomechanical issues, diminished cardiorespiratory fitness, and a markedly higher prevalence of type-2 diabetes (up to four-hold higher) [3,5]. Consequently, early intervention and continuous monitoring of weight status are widely recommended in this population [6].

In individuals with DS, the increased risk of developing overweight or obesity is thought to be due to a combination of general risk factors, and those specific to this population, including: elevated concentrations of leptin, low resting energy expenditure, hypotonia, low physical activity levels, and the presence of comorbid conditions such as hypothyroidism that may predispose to weight gain [3].

However, despite the well-documented burden of obesity and its associated complications in DS, important gaps remain regarding the characterization of body composition and its relationship with lifestyle factors across different age groups in this population.

Cognitive deficits in individuals with DS may also have an impact on their eating behaviors and influence their food choices, resulting in poor dietary quality and subsequent weight problems [3,7,8]. Furthermore, certain medications utilized to address conditions associated with DS, including steroids, anti-inflammatory and antipsychotic drugs, have the potential to influence appetite and contribute to body weight gain [2,4]. Overall, the increased risk of obesity among individuals with DS is likely due to a complex interplay between environmental, behavioral, and biological influences. The dietary recommendations for individuals with DS generally align with those for the general population. Maintaining a balanced and nutritious diet is essential, but specific macronutrient or micronutrient intake and distributions have not been studied for DS individuals, with lack of data on dietary intake during the at-risk period for obesity (teenage years and early adulthood). Instead, it is recommended to adhere to national dietary guidelines, adapting them as necessary to accommodate individual needs [6].

The determination of energy requirements in individuals with DS can pose a challenge owing to various factors, such as the severity of intellectual disability, mobility, age, medications, and feeding issues. Predictive equations, frequently based on height and weight, have the potential to underestimate energy requirements, particularly in individuals with DS. The National Academy of Medicine has developed a set of equations that can be utilized to accurately assess energy requirements for adolescents with diabetes, incorporating distinct formulas for individuals with healthy weight and overweight/obese individuals [9].

The EU H2020-funded Go-DS21 project (Grant agreement ID: 848077) aims to identify shared mechanisms of comorbidity between obesity and intellectual disability in DS.

For the human workstream of this project, we aimed to investigate lifestyle and other factors that contribute to increased body weight in DS individuals, with a particular focus on the role of dietary constituents and overall diet quality. By examining dietary quality, macronutrient intake, and physical activity in a multicenter European context, the study also considers how cultural and environmental factors may contribute to body weight, body composition, and general health in this population. Conducted across three recruiting clinical sites in different European countries, this design allows for the exploration of these influences and increases the relevance and generalizability of the findings. Based on this rationale, we hypothesize that higher dietary quality, adequate macronutrient intake, and greater physical activity levels may be associated with a healthier anthropometric and body composition profile and potentially better cognitive outcomes in individuals with DS.

## 2. Materials and Methods

### 2.1. Study Design

Non-drug, prospective, cross-sectional, and multicenter study of individuals with DS. Participants were evaluated at three clinical research sites, members of the GO-DS21 consortium and recognized as European centers of excellence in the field of Down syndrome: one at King’s College London (KCL, London, UK), one at Hospital del Mar Research Institute (HMRI, Barcelona, Spain), and one at the Institut Jérôme Lejeune (IJL, Paris, France). The overall objective of the Go-DS21 project is to understand the impact of gene overdosage in comorbidities associated with DS, particularly intellectual disability and obesity. In the present study, we aimed to determine the role of lifestyle factors, including diet, nutrient intake, and physical activity levels as risk factors for overweight and obesity in the DS population. Individuals within three categories of body mass index (BMI) were considered: normal weight (BMI 18.5–24.9), overweight (BMI 25–29.9), and obese (BMI > 30). All data presented in this work were collected at a baseline visit, which included the signature of informed consent, the report of medical history, a medical exploration, biological samples collection, neuropsychological, functional, and lifestyle (dietary and physical activity) data collection, as well as anthropometric and body composition data collection.

### 2.2. Research Participants

**Figure 1:**
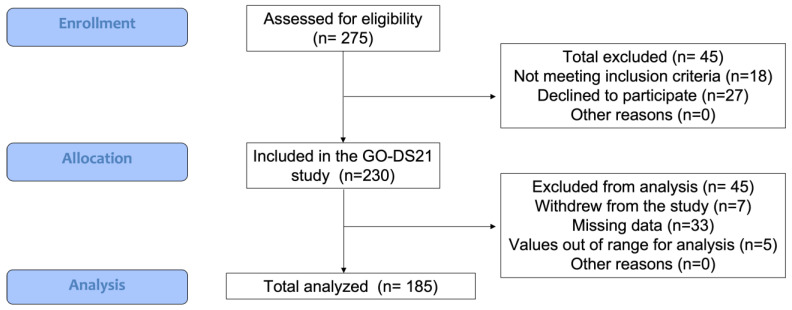
Consort diagram. Flow of participants through the study, indicating the total sample recruited, the total sample analyzed, and the reasons for exclusions.

Two hundred and thirty participants (*n* = 230) were enrolled in the “Elucidating age-related comorbidity patterns in Down syndrome” work package of the Go-DS21 project. From the whole sample, one hundred and eighty-five subjects (*n* = 185) with DS (full trisomy 21, confirmed by karyotyping) including children/adolescents (12–18 years old), young adults (19–35 years old), and adults (36–45 years old) of both genders (44.9% females) were considered for this analysis at the three participating clinical sites (Figure 1, consort diagram). From the 185 subjects analyzed, a subsample of 48 participants from Barcelona was further studied to investigate the relationship between the level of intellectual disability and the dietary and body composition measures considered in the study. Recruitment took place between June 2020 and August 2024. Participants were recruited using multiple strategies, including existing databases of individuals who had previously participated in research studies at the recruiting centers, informational sessions, and collaboration with organizations and associations of the Down syndrome community. At the baseline visit, participants, parents, and/or legal guardians were informed of the details of the study and gave their informed written consent before taking part.

### 2.3. Inclusion and Exclusion Criteria

Participants selected for the study were deemed eligible based on the following criteria: (a) males and females between the ages of 12 and 45; (b) a confirmed diagnosis of DS (full trisomy 21; based on medical records or karyotype results); (c) a parent or legal guardian/representative and caregiver who was willing to provide written informed consent, and who was available to accompany the participating subjects to the clinical visits when necessary; (d) subjects who were able to adhere to the procedures and understand basic instructions.

Participants with medical history of a confirmed dementia diagnosis were excluded from the study, as well as participants with unstable concomitant diseases, comorbid conditions, or any clinically significant finding at screening that could interfere with the conduct of the study or that would, in the opinion of the investigator, lead to an unacceptable risk to the subject in this study. Furthermore, participants were excluded if they had participated in other clinical studies within the previous three months or if they presented a mosaic trisomy 21, partial trisomy 21, or translocation, confirmed by karyotyping.

### 2.4. Ethics Statement

The clinical studies adhere to the ‘Ethical Guidelines for Biomedical Research on Human Participants’ to safeguard the dignity, rights, safety and well-being of all the participants in the study (WMA Declaration of Helsinki, reviewed in Helsinki, Finland, October 2024), the guidelines of the World Health Organization, and the EU legislation on personal data, namely, Regulation (EU) No 2016/679 of the European Parliament and of the Council of 27 April 2016 on Data Protection (RGPD). The study protocol was approved by each local institutional review board (IRBs) (NRES and HRA in London, Parc de Salut Mar Clinical Research Ethics Committee CEIm-PSMAR in Barcelona, Comité de Protection des Personnes Sud Méditerranée III in Paris). The description of the protocol can be found in ClinicalTrials.gov (NCT05310552).

### 2.5. Dietary Assessment

#### 2.5.1. Food Frequency Questionnaire (FFQ)

In order to assess the usual dietary intake of the participants during the preceding year, we used standard FFQs, each one tailored to a specific country: the French MetaCardis FFQ for France (159 items) [10], the EPIC-Norfolk FFQ for the UK (130 items) [11], and the PREDIMED FFQ for Spain (140 items) [12].

Each FFQ comprised a limited number of foods and beverages that reflected the typical foods consumed in each country, with common general categories including milk and dairy products, eggs, meat and fish, vegetables, fruits, legumes and grain products, oils and fats, sweets and snacks, as well as fast food or ultra-processed products. FFQs were answered by parents or caregivers of study participants filling a web-based version of the questionnaire using the EUSurvey platform (https://ec.europa.eu/eusurvey (accessed on 27 February 2021); version 1.5.1). The questionnaire was designed to be completed independently, and participants were provided with detailed instructions on how to complete it; however, when needed, it could also be completed at the study center with the assistance of a trained study professional. The frequency of food consumption for each food or beverage ranged from never or rarely to more than six times per day and its consumption was considered during the past year. Participants with questionnaires with some unanswered items were excluded from the analysis.

#### 2.5.2. Nutrient Intake Calculation

The nutrient intake was estimated using data from the FFQ, using established methods: for each food item, the consumption frequency was multiplied by its nutrient content per standard portion. The nutrient content was obtained from the official nutritional composition tables of each participating country. These values were then summed to calculate the total nutrient intake. Individuals with implausible energy intake were excluded to guarantee data quality. Specifically, women who reported having energy intakes exceeding 5000 kcal or below 500 kcal, and men who reported intakes exceeding 6000 kcal or below 500 kcal were excluded from the analysis [13].

#### 2.5.3. Healthy Eating Index 2020

The Healthy Eating Index 2020 (HEI-2020) is a measure of overall dietary quality, independent of diet quantity, developed by the United States Department of Agriculture’s (USDA) Center for Nutrition Policy and Promotion [14]. Thirteen components are considered with scores ranging from 0 to 100. Higher scores are related to healthier diets. The HEI-2020 was calculated for each participant.

### 2.6. Physical Activity Assessment

The physical activity (PA) level was evaluated using the Spanish short version of the Minnesota Leisure Time Physical Activity Questionnaire (VREM) [15], a questionnaire that assesses the level of physical activity, sports, and leisure according to energy expenditure (EE). The Spanish short version of the questionnaire was translated into French and English. The VREM questionnaire was answered by participants and their parents and/or caregivers, with support from a trained study professional who assisted with questionnaire completion and clarified any questions when needed.

Participants were categorized based on their total energy expenditure during their leisure time for a period of 14 days, which was calculated in METs (metabolic equivalent of the task). The following categories were considered: sedentary (EE < 1250 METS-min/14 days), moderately active (EE between 1250–2999 METS-min/14 days), active (EE between 3000–4999 METS-min/14 days), and very active (EE > 5000 METS-min/14 days).

### 2.7. Anthropometric and Body Composition Analysis

Body composition was assessed through Bioelectric Impedance Analysis using the same Tanita Segmental Body Composition Monitor Model BC-601 (Tanita Corporation, Tokyo, Japan) at all participating sites, ensuring methodological consistency. Anthropometric and body composition parameters were obtained automatically by the device following a standardized measurement protocol. The parameters evaluated for adult participants included: weight (kg), body fat (%), muscle mass (kg), bone mass (kg), total body water (%), visceral fat level, and Body Mass Index (BMI, Kg/m2). For children (participants under 18 years old) only weight, % of body fat, and BMI were obtained. The estimation of weight category for subjects under 18 was done using the z-scores/standard deviations thresholds based on BMI and year of birth (from the World Health Organization; https://www.who.int/tools/growth-reference-data-for-5to19-years/indicators/bmi-for-age (accessed on 10 March 2023), as follows—normal weight: −2 < z-score < 1; overweight: 1 < z-score < 2; obese: z-score > 2. The waist and hip circumferences were also evaluated, using a tape measure in all participants (in centimeters). All professionals doing these measurements received prior training to ensure consistency across sites, and measurements were performed following a common protocol to reduce variability between centers.

### 2.8. Cognitive Assessment

Leiter International Performance Scale, 3rd edition [16].

Individually administered, the Leiter-3 is a non-verbal test used to evaluate the Intellectual Quotient (IQ). It has been designed for individuals between ages 3 and 75 years old and it evaluates non-verbal cognitive, attentional, and neuropsychological abilities. The final non-verbal IQ score and the related cognitive sub-scores (figure–ground, form completion, classification/analogies, and sequential order) were recorded.

### 2.9. Statistical Analysis

Descriptive statistics were calculated for study variables, using absolute and relative frequencies (%) for categorical variables, and mean and standard deviation (SD) for numeric variables. Linear mixed models were used to analyze the associations between the variables of interest and weight categories (normal weight, overweight, obesity), recruiting centers and sex. These models were adjusted for the potential confounders sex and age, and the study center was included as a random effect. The ‘Underweight’ category was excluded from some analyses due to its small sample size (*n* = 7). In the case of statistically significant differences between weight categories, pairwise post hoc comparisons were conducted using the Tukey test. To quantify the differences between the three study centers regarding dietary intake estimates from the Food Frequency Questionnaire, Cohen’s f was used. According to Cohen, values above 0.25 and 0.40 indicate medium and large differences, respectively [17]. In addition, with the subsample from the study center in Barcelona, linear mixed models were applied to assess the associations between the variables of interest and the IQ, adjusting for age and sex.

Statistical analyses were performed in R (Version 4.4.1, R Foundation for Statistical Computing, Vienna, Austria) using the packages nlme [18] and multcomp [19] with a significance level of *p* < 0.05.

## 3. Results

### 3.1. Descriptive Demographics and Clinical Data of Participants

Socio-demographic data and clinical parameters of all participants involved in the analysis are shown in Table 1.

One hundred eighty-five participants from 3 clinical sites were included in this study. The sample size varied slightly across centers, with a larger number of participants from the IJL (*n* = 88, 47.6% of the total sample). However, the samples from the three sites had very similar distributions in terms of age (mean age: KCL = 24.0 [8.7], HMRI = 26.9 [8.7], IJL = 23.6 [10.2]) and sex (percentage of females: KCL = 44.7%, HMRI = 48%, IJL = 43.2%). The distribution of participants by age, considering three age ranges (adolescents aged 12–18 years, young adults aged 19–34 years, and adults aged 35–45 years), is shown in Figure S1. Concerning weight categories, the proportion of participants with normal weight was larger at IJL (56.8%) compared to KCL and HMRI (42.6% and 42%, respectively). Furthermore, the underweight group was poorly represented in the total population with only 7 participants belonging to this weight category group (KCL *n* = 0, HMRI *n* = 1, IJL *n* = 6).

Additionally, the proportion of participants with moderate intellectual disability (ID) was higher at IJL (76.8%), while KCL and HMRI had a substantially higher proportion of individuals with mild ID (57.4% and 58.0%, respectively). Common comorbid conditions identified in the sample that may have an impact on the dietary profile are also reported (Table S1).

### 3.2. Demographic and Dietary Variables with an Impact on the BMI

**Figure 2:**
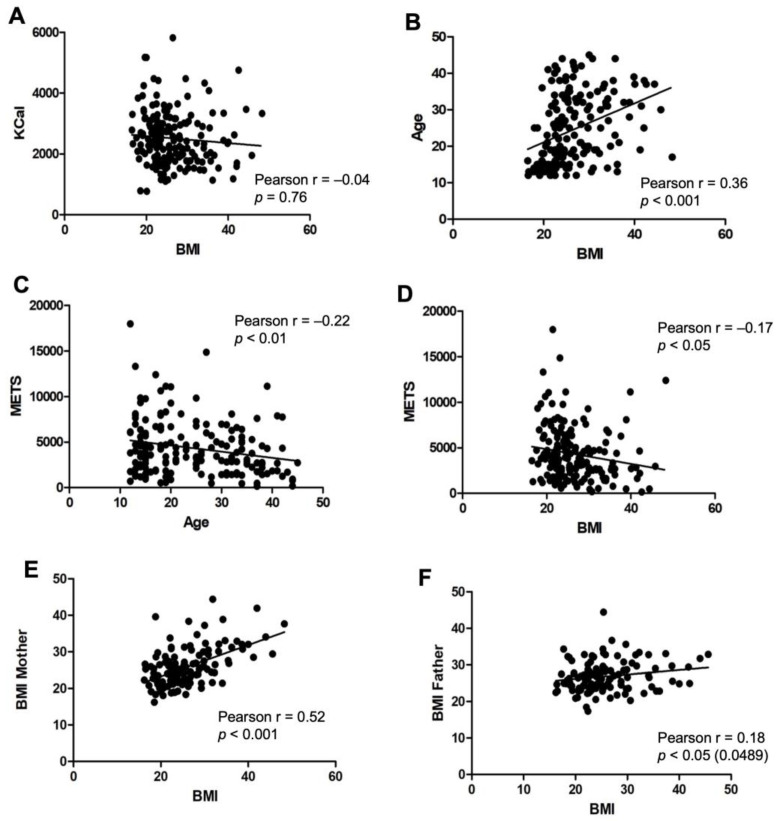
Impact of demographic and dietary variables on the body mass index. (**A**) No significant correlations were found between participants’ BMI and their total caloric intake (kCal); (**B**) significant positive correlations were observed when compared to age, with higher values of BMI as participants get older. Physical activity showed negative correlations both with BMI (**C**) and age (**D**). BMI of participants compared with parental BMI demonstrated significant positive correlations with both their mother’s BMI (**E**) and their father’s BMI (**F**). Each dot represents an individual participant’s data, and the solid line indicates the linear regression line. Significant differences were considered when *p* < 0.05.

The impact of different variables on the BMI was examined. No correlation was found between total energy intake and BMI (r = −0.06, *p* = 0.39) (Figure 2A), suggesting that body weight is independent of the total caloric consumption. A significant positive correlation was observed between age and BMI (r = 0.34, *p* < 0.001), indicating that older participants have higher body weights (Figure 2B). A negative correlation was found between physical activity and BMI (r = −0.18, *p* < 0.05) (Figure 2C), suggesting that lower levels of physical activity are associated with higher BMI. Moreover, the level of physical activity negatively correlated with age (r = −0.22, *p* < 0.01) (Figure 2D), with older participants exhibiting lower levels of exercise. Parental BMI was also positively correlated with participant BMI (Figure 2E,F), with maternal BMI showing a stronger correlation (*n* = 136; r = 0.53, *p* < 0.001) compared to paternal BMI (*n* = 127; r = 0.15, *p* < 0.05).

### 3.3. Anthropometric and Body Composition Analysis

**Figure 3:**
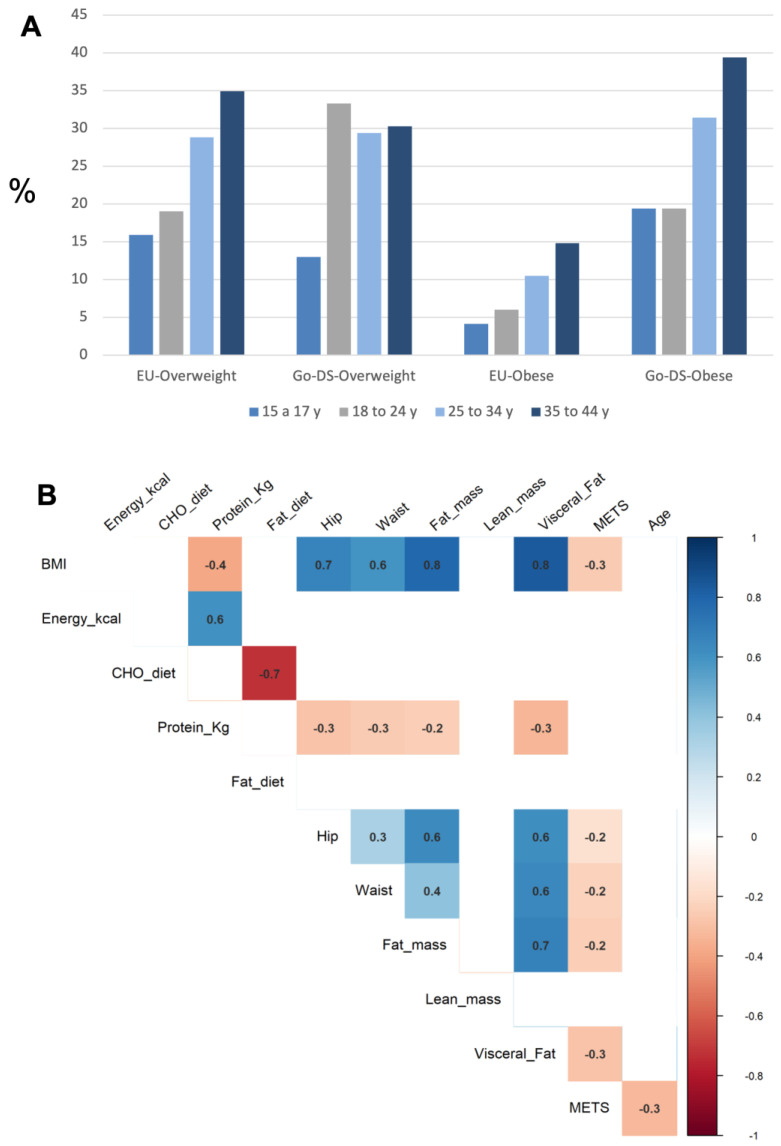
Overweight, obesity, and body composition measures in GO-DS21 participants. (**A**) Prevalence of overweight and obesity analyzed in comparison to data from European general population across different age ranges. (**B**) Body composition parameters analyzed in adult participants showed that excessive body weight is positively correlated with percentage body fat, visceral fat, and hip and waist circumferences. All parameters were negatively correlated with reported physical activity. The color of the comparisons indicates the *p*-value for statistical significance, with darker shades indicating a more significant correlation (dark blue for positive correlations, dark red for negative correlations).

Forty-nine percent of the participants exhibited a high BMI (above 25 kg/m^2^), being categorized as participants with overweight or obesity. Compared with EU general population data statistics (data available from 15 to 44 years old), DS participants may have a higher prevalence of obesity than the general population [20] in four different age ranges: 15–17 years, 18–24 years, 25–34 years, and 35–44 years (Figure 3A). However, in adolescents (12–14 years), the prevalence of overweight and obesity in the EU general population has been reported to be 21% [21], similar to the prevalence found in our sample of adolescents with DS, which was 24%.

Further body composition analysis of adult participants (*n* = 95) revealed that this excess of weight correlated with adiposity parameters such as the percentage of body fat (r = 0.79, *p* < 0.001), visceral fat (r = 0.83, *p* < 0.001), and hip and waist circumferences (r = 0.67, *p* < 0.001; r = 0.60, *p* < 0.001, respectively) (Figure 3B). All the adiposity parameters were negatively correlated with the reported physical activity, while muscle mass showed no correlation with physical activity or any other anthropometric/body composition parameter. Interestingly, the energy intake was not associated with BMI categories or adiposity parameters.

Abdominal obesity, defined by the waist-to-hip ratio (>0.85 in women and >0.90 in men) as described by the WHO, was identified in 56.3% of the study population, and 17.6% showed ratios above 1, indicating a notably higher risk of health issues.

### 3.4. Dietary Intake Profile According to BMI Category

Dietary intake of nutrients, micronutrients, and a diet quality index were evaluated across BMI categories (underweight, normal weight, overweight, and obese), excluding participants with underweight from statistical analysis due to low sample size. The results are presented in Table 2. No significant differences were observed in the estimated intake of nutrients and micronutrients between body weight categories, except for protein intake. A consistently significant relationship between decreasing protein consumption and increasing BMI was observed, with the following pairwise comparisons: normal weight vs. people with overweight *p* = 0.051; normal weight vs. people with obesity *p* < 0.001; people with overweight vs. people with obesity *p* = 0.005; that is, higher protein intake in normal-weight individuals and lower consumption in obese individuals. These findings were consistent when analyzed separately by clinical sites (Figure S2A–C).

**Figure 4:**
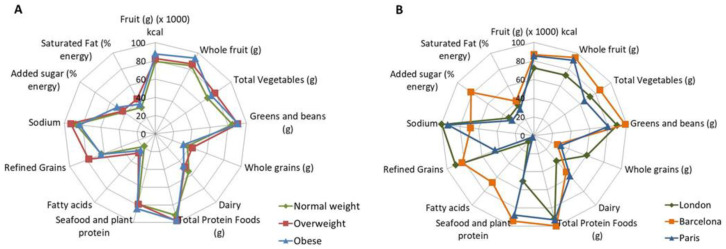
HEI-2020. The Healthy Eating Index (HEI-2020) was analyzed to study quality of diet independent of diet quantity. (**A**) Similar results were obtained in the consumption of the 13 distinct constituents considered while (**B**) significant differences in the quality of diet were observed when the analysis was performed between the different countries involved in the study. Adjustments were performed in data analysis considering age, gender, and center.

Diet quality was assessed using the HEI-2020, based on 13 dietary components. The overall profile of the different food groups’ consumption was similar between body weight categories (Figure 4A) showing a similar food intake pattern. Individuals with overweight tended to have higher total HEI scores than both individuals with normal-weight and individuals with obesity. However, these differences were not statistically significant (overweight vs. normal weight: *p* = 0.088; overweight vs. obese: *p* = 0.053).

### 3.5. Dietary Intake Profile According to Recruiting Centers

Dietary intake estimation of different nutrients, micronutrients, and HEI-2020 score was analyzed considering the 3 clinical sites. Table 3 outlines the main differences. Disparities in dietary intake profiles were found in total energy intake (kCal/d, Figure S3A) and in several macronutrients including total carbohydrates (f > 0.40), proteins (f > 0.40), total fat (f > 0.40), monounsaturated fatty acids (f > 0.40), polyunsaturated fatty acids (f > 0.40), cholesterol (f > 0.40), fiber (f > 0.25), and sugar (f > 0.25), (Figure S3B–I). Only small differences were found for saturated fatty acids (f = 0.09) (Figure S3J). Significant differences were also identified in micronutrient intake.

HEI-2020 scores differ notably among countries, with participants in Barcelona showing the highest diet quality (mean HEI-2020 = 70.2 [9.9]), followed by London (mean HEI-2020 = 59.0 [8.4]), and Paris having the poorest quality (mean HEI-2020 = 54.1 [8.6]). Different dietary constituent profiles were also observed across clinical sites (Figure 4B).

### 3.6. Sex Differences in Weight and Dietary Parameters

Body composition and estimated dietary intake parameters were compared between genders. As expected, women had higher BMI, body fat percentage, hip circumference, but lower muscle mass than men. Visceral body fat and waist circumferences were not statistically different between genders.

Regarding dietary intake, men had a higher energy intake than women (mean kcal/day = 2662 [907] vs. 2406 [885]; *p* = 0.014), with no significant differences in most macro- and micronutrients. However, women had a higher diet quality, reflected by higher HEI-2020 scores (mean HEI = 61.6 [10.4] versus 58.1 [11.4]; *p* = 0.008), as well as a significant higher consumption of added sugar, whole fruit, whole grain, total protein foods, seafoods, and plant protein (Table S2).

### 3.7. Dietary Intake Profile, Anthropometric Measures, and Body Composition According to IQ in Participants from Barcelona

**Figure 5:**
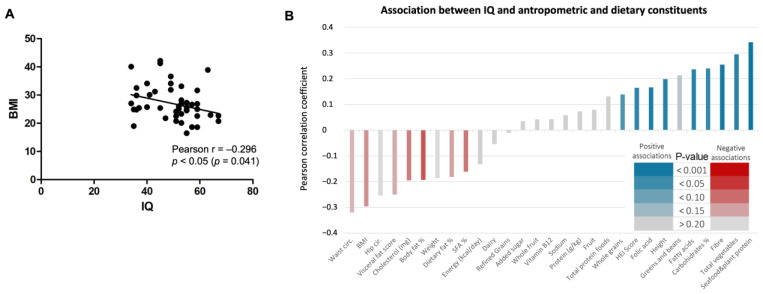
Relationship between IQ, BMI and dietary, anthropometric, and body composition parameters. (**A**) A negative significant correlation was observed between IQ and BMI in a subsample of participants from Barcelona. (**B**) The association between IQ and anthropometric, body composition, and dietary constituents was represented as Pearson correlation coefficients (r). Each vertical bar represents the correlation between IQ and one specific variable. Bars are ordered by the magnitude and direction of the correlation, from the strongest negative associations on the left to the strongest positive associations on the right. In figure A each dot represents an individual participant’s data, and the solid line indicates the linear regression line. Significant differences were considered when *p* < 0.05. The statistical significance is represented by a color gradient, where darker shades of red (negative) or blue (positive) indicate stronger statistical significance. The specific scale for these color coded *p*-values can be seen in the legend of Figure 5B.

A sub-study in Barcelona participants *(n* = 48), showed a significant negative correlation between IQ and BMI (r = −0.3, *p* < 0.05) (Figure 5A), indicating that higher weight categories were associated with lower IQ scores.

A more in-depth analysis revealed interesting associations between IQ and various anthropometric, body composition, and dietary constituents (Table S3). Higher IQ scores were positively associated with the consumption of sea food and plant protein, total vegetables, fiber, fatty acids, and folic acid, as well as with height. Conversely, negative associations were found between higher IQs and body fat percentage, cholesterol or waist circumference (Figure 5B). Altogether, despite the limitations of cross-sectional data, these results suggest that individuals with DS with higher IQ tend to have healthier diets and better physical health.

## 4. Discussion

We reported the outcomes of an observational study conducted on the framework of the Go-DS21 project in a DS cohort from the UK, Spain, and France, aged 12–45. We examined the causes and main determinants of overweight/obesity in DS, with a particular focus on dietary intake. Regarding dietary factors, excess weight was inversely associated only with protein intake, while no association was observed with caloric intake or overall diet quality. Diet profiles differed significantly among the participating countries, reflecting variations in national dietary patterns.

Previous studies have shown that individuals with DS had a higher prevalence of overweight/obesity compared to the general population. Body weight results revealed that approximately half of our participants fell within the normal weight range, which also confirmed a higher prevalence of overweight/obesity [3,4]. Although this study was not designed to estimate the prevalence of obesity in DS, compared with the general population, the percentage of participants with a BMI higher than 25 in our study was higher [20,21]. A moderately positive correlation was observed between age and BMI, with older individuals more likely to exhibit higher BMI, suggesting a cumulative effect of weight gain over time, at least into mid-life, and highlighting the importance of early monitoring and interventions throughout the lifespan [22]. As anticipated, a significant correlation was observed between excessive body weight and certain adiposity parameters, including body fat, visceral fat, and hip and waist circumferences. Notably, the high percentage of participants presenting abdominal obesity may be explained by the characteristic body fat distribution of this population [3].

No significant correlation was observed between energy intake and BMI. This contradicted our hypotheses and suggests that body weight in DS individuals can be affected by additional factors independently of the energy consumed. Negative correlations were found between physical activity levels and BMI and between reported physical activity and all adiposity parameters, highlighting its importance in weight management. Some features of DS (such as hypotonia and hypothyroidism) and the increased rates of obesity in this population are associated with decreased energy expenditure and reduced physical activity [6,23]. Given our results, the promotion of appropriate recommendations for physical activity is as important in DS as in the general population. Although the guidelines for physical activity may be similar for DS and the general population, certain adaptations may be required. For example, low-intensity physical activities within the daily routines can be advantageous for weight management and adiposity reduction, especially for individuals with limited ability to engage in more vigorous exercise [6,24]. Interestingly, participants’ BMI also correlated with parental BMI, particularly maternal BMI, an observation widely described in the general population [25]. This may be attributed to a combination of genetic, shared family environmental factors, and possibly intrauterine programming and supports targeting the family/environmental setting in weight-loss interventions [26]. Altogether, these results demonstrate the complex interplay between genetic, demographic, and environmental factors on body weight in DS.

The average energy intake across weight categories exceeds or falls within the upper range of the European Food Safety Authority (EFSA) energy recommendations in the general population [27]; however, these recommendations may need to be adapted for the DS population. Macronutrient and micronutrient intake did not significantly differ across body weight categories except for protein, which was observed to decrease with increasing BMI across all clinical sites. Dietary protein may play a multifaceted role in weight loss and maintenance potentially influencing key metabolic targets that regulate body weight including satiety, thermogenesis, energy efficiency, and body composition [28]. High-protein diets have been demonstrated to promote weight loss and prevent weight regain after weight loss, increasing energy expenditure [29]. This mechanism involves both a higher thermogenic effect of proteins compared to other nutrients and the preservation of fat-free mass, which contributes to maintaining resting energy expenditure despite weight loss. Complete proteins, containing all essential amino acids, lead to greater increases in energy expenditure compared to lower-quality proteins [30].

Our findings suggest that DS individuals might benefit from increasing good-quality protein intake, although that hypothesis requires further investigation. This could be particularly beneficial for addressing two mechanisms contributing to a higher incidence of body fat. Firstly, DS has been associated with alterations in leptin metabolism, leading to an inherent genetic predisposition for increased leptin resistance [31], which could interfere with satiety regulation. A high-protein diet could potentially promote satiety through a different pathway, compensating for leptin resistance and improving satiety regulation. Additionally, protein intake may induce an elevation in energy expenditure, which could help to balance the lower resting energy expenditure observed in DS individuals. Further research is needed to confirm this observation and to investigate the impact of transitioning to a high-protein diet on weight management in the DS population.

The HEI-2020 score measures diet quality. While no significant differences in diet quality were observed between weight categories, notable variations emerged among countries. Moreover, significant differences were found regarding total energy intake and in almost all macro- and micronutrients analyzed. These country-specific disparities can be influenced by a complex interplay of cultural, environmental, and contextual factors, including food preferences, ingredient availability, culinary/cooking traditions, national dietary guidelines, and nutrition education policies. For example, the higher diet quality observed in Barcelona may reflect greater adherence to traditional Mediterranean dietary patterns, characterized by higher consumption of fruits, vegetables, legumes, whole grains, and olive oil, which have been consistently associated with favorable health outcomes and obesity prevention in the general population [32,33]. In contrast, variations in dietary patterns in the other participating countries may relate to different habitual food choices, levels of food processing, or macronutrient distributions shaped by local food environments. The differences among countries highlight the importance of considering cultural and geographical factors when assessing dietary intake and promoting healthy eating habits.

These findings underscore the importance of interpreting dietary intake data within its cultural and geographical context and suggest that nutritional recommendations and interventions for individuals with DS may need to be tailored to country-specific environments rather than adopting a common approach. It is worth noting that these comparisons were made among only three European countries, highlighting the need for even greater caution when generalizing findings to other populations with different cultural, socioeconomic, and dietary contexts.

Despite variations among countries, the HEI-2020 values obtained in our DS cohort do not significantly differ from those reported in the general population [34]. This observation challenges the misconception that individuals with DS have poor dietary habits. Nonetheless, although the dietary quality profile is similar, it remains suboptimal, with several aspects that could benefit from targeted improvement.

We also evaluated the influence of diet, anthropometric, and body composition measurements on IQ within a subsample of participants from Barcelona, showing that a higher BMI is correlated with lower IQ level. A consistent association between IQ and healthy dietary patterns was found, including high-quality protein sources, vegetable intake, and whole grains. Additionally, a healthy lipid profile—specifically, the ratio of polyunsaturated (PUFAs) and monounsaturated fatty acids (MUFAs) to saturated fatty acids (SFAs)—was associated with IQ. These food groups are nutrient-dense and rich sources of omega-3 and omega-6 fatty acids, phenolic compounds, and antioxidants, essential to support brain health and cognitive function.

A positive association worth noting is that between IQ and height, a relationship previously described in the DS population [35]. Conversely, lower IQ was associated with adiposity parameters related to excess body weight and low-quality fat intake. These observations stem from a limited sample size and are merely associative, so definitive conclusions cannot yet be drawn. Therefore, the findings regarding cognitive outcomes should be considered preliminary, and any practical recommendations should be approached with caution, highlighting the importance of future longitudinal and interventional research to verify these associations. Nonetheless, these findings highlight that a diet rich in these nutrients together with weight control may potentially support cognitive functioning in DS individuals. In addition, emerging evidence suggests that engaging in regular physical activity may support cognitive function in adults with DS [36], highlighting the importance of the combination of different lifestyle factors to improve cognitive impairment.

Some limitations of this study should be acknowledged. The broad age span of participants (including children and adolescents) may have introduced heterogeneity in lifestyle behaviors, body composition, and dietary habits, potentially influencing the observed associations. However, this wide age range also provides a comprehensive transversal characterization of individuals with DS across different life stages. In addition, although standardized procedures were applied, some assessments required support from caregivers or trained professionals, which may have affected data accuracy. Greater consideration should also be given to potential sources of bias, including dietary misreporting, reverse causality, and the limitations inherent to the cross-sectional design, which are widely acknowledged methodological challenges in clinical research involving human participants. Furthermore, as dietary patterns are strongly shaped by sociocultural context, the results derived from the participating countries should be interpreted with caution and may not be directly extrapolated to other populations.

Nevertheless, the consistently elevated prevalence of overweight and obesity reported in individuals with DS across diverse regions highlights the global relevance of addressing modifiable lifestyle factors in this population. Our findings clearly underscore that individuals with Down syndrome do not constitute a homogeneous group, reinforcing the need to consider this variability when designing and implementing personalized lifestyle interventions, at different levels.

This study is the first to compare food intake and physical activity and its association with BMI in individuals with DS across different European countries. We identified cross-cultural similarities and differences in both population characteristics and dietary patterns. However, the study also has some limitations. The sample may not be representative of all individuals with DS, so we cannot estimate overweight and obesity rates or prevalence and have therefore focused on factors associated with weight in the study population. Finally, as a cross-sectional observational study, the GO-DS21 study is unable to definitively establish a causal relationship, as reverse causation remains a potential confounding factor.

## 5. Conclusions

In conclusion, our findings provide valuable insights into the complexity and underlying causes of the excess weight commonly found in DS, as well as potential strategies for effective interventions. While dietary quality in subjects with DS appears comparable to the general population, the inverse association between protein intake and BMI suggests a potential role for a higher protein intake in weight management. Additionally, promoting physical activity remains essential for individuals with DS. In that sense, involving families in dietary and physical activity interventions is crucial for long-term adherence and success. Moreover, preliminary data suggest that a higher-quality diet may be linked to better cognitive functions.

Further research is warranted to explore the impact of a higher protein diet and to tailor dietary and physical activity interventions to the specific needs of individuals with DS, considering their unique metabolic characteristics and sociocultural variations between different countries.

## Figures and Tables

**Table 1:** Demographics characteristics of study participants according to the recruiting site (London, Barcelona, and Paris).

	London *n* = 47	Barcelona *n* = 50	Paris *n* = 88
Age: mean (SD)	24.0 (8.7)	26.9 (8.7)	23.6 (10.2)
Sex: *n* (%)			
Female	21 (44.7%)	24 (48.0%)	38 (43.2%)
Male	26 (55.3%)	26 (52.0%)	50 (56.8%)
BMI: mean (SD)	27.1 (5.1)	27.1 (6.3)	25.7 (6.8)
Weight category: *n* (%)			
Underweight	0 (0.0%)	1 (2.0%)	6 (7.8%)
Normal weight	20 (42.6%)	21 (42.0%)	46 (52.3%)
Overweight	16 (34.0%)	14 (28.0%)	17 (19.3%)
Obese	11 (23.4%)	14 (28.0%)	19 (21.6%)
ICD_10: *n* (%)			
Mild ID	27 (57.4%)	29 (58.0%)	17 (20.7%)
Moderate ID	17 (36.2%)	16 (32.0%)	63 (76.8%)
Severe ID	3 (6.4%)	5 (10.0%)	2 (2.44%)
Energy expenditure in METS (in 14 days): mean (SD)	4308 (2978)	3904 (2277)	4485 (3283)
Physical activity: *n* (%)			
Sedentary	6 (13.0%)	3 (6.00%)	9 (10.2%)
Moderately active	13 (28.3%)	19 (38.0%)	27 (30.7%)
Active	12 (26.1%)	15 (30.0%)	22 (25.0%)
Very active	15 (32.6%)	13 (26.0%)	30 (34.1%)

**Table 2:** Dietary intake estimates from the Food Frequency Questionnaire according to BMI category.

	Underweight *n* = 7	Normal Weight *n* = 87	Overweight *n* = 47	Obese *n* = 44	*p* Value
**BMI**	17.3 (0.72)	22.1 (1.81)	27.2 (1.50)	35.8 (4.70)	<0.001
**Total energy (kcal/d)**	2674 (700)	2582 (945)	2562 (886)	2442 (889)	0.790
**Nutrients**					
Total carbohydrates (% TEI)	47.1 (6.60)	48.8 (8.31)	49.5 (8.69)	47.4 (8.42)	0.529
Protein (g/weight)	2.92 (0.94)	2.17 (0.91)	1.73 (0.73)	1.31 (0.47)	**<0.001**
Total fat (% TEI)	35.2 (5.95)	33.7 (7.34)	34.2 (7.50)	34.9 (7.31)	0.792
SFA (% TEI)	16.3 (4.26)	14.1 (3.68)	13.4 (4.86)	13.2 (2.81)	0.695
MUFA (% TEI)	11.8 (3.96)	12.5 (5.34)	14.0 (6.88)	13.6 (5.32)	0.684
PUFA (% TEI)	4.47 (1.95)	4.69 (2.05)	5.58 (3.51)	5.04 (1.37)	0.178
Cholesterol (mg/d)	425 (154)	348 (194)	336 (174)	347 (167)	0.978
Fiber (g/d)	35.2 (19.3)	33.0 (19.4)	35.7 (16.7)	30.7 (13.6)	0.161
Sugar (g/d)	164 (86.0)	133 (70.8)	133 (56.0)	114 (46.2)	0.325
**Micronutrients**					
Folic acid (µg/d)	631 (331)	522 (307)	567 (263)	479 (224)	0.059
B12 (µg/d)	4.62 (2.11)	4.00 (2.66)	3.71 (2.56)	3.41 (2.36)	0.621
Sodium (mg/d)	2455 (906)	2695 (1367)	2457 (1146)	2703 (1179)	0.641
Potassium (mg/d)	4611 (1457)	4552 (1857)	5039 (1732)	4351 (1387)	0.111
Calcium (mg/d)	1135 (230)	1305 (627)	1398 (736)	1137 (394)	0.195
Magnesium (mg/d)	397 (138)	419 (170)	461 (178)	389 (128)	0.060
Iron (mg/d)	14.2 (5.54)	13.7 (6.14)	15.2 (7.82)	13.4 (5.86)	0.224
**Food group composition (Standardized per 1000 kcal)**					
Fruit	205 (104)	142 (93.5)	182 (132)	171 (86.3)	0.121
Whole fruit (excluding juices)	118 (62.5)	114 (90.9)	133 (106)	136 (80.5)	0.678
Total vegetables	192 (70.3)	183 (119)	219 (121)	184 (88.5)	0.113
Greens and beans	67.3 (59.3)	63.5 (50.8)	77.9 (46.3)	71.5 (48.4)	0.550
Whole grains	8.86 (13.3)	24.5 (34.3)	24.0 (27.7)	19.6 (28.7)	0.383
Dairy	160 (57.9)	174 (110)	164 (149)	143 (93.9)	0.590
Total protein foods	111 (65.8)	103 (56.3)	111 (43.6)	118 (47.3)	0.845
Seafood and plant protein	38.7 (44.9)	30.3 (24.1)	35.6 (25.4)	35.9 (24.8)	0.330
Fatty acids profile *	1.06 (0.43)	1.29 (0.58)	1.51 (0.61)	1.46 (0.56)	0.172
Refined grains	81.3 (14.8)	71.7 (47.7)	56.3 (30.9)	73.8 (46.8)	0.100
**Diet Quality**					
Healthy Eating Index 2020	53.5 (4.63)	57.9 (11.6)	63.4 (10.2)	60.2 (10.7)	**0.039**

D: Day; TEI: Total Energy Intake; SFA: Saturated Fatty Acids; MUFA: Monounsaturated Fatty Acids; PUFA: Polyunsaturated Fatty Acids * Fatty acids profile: (PUFAs + MUFAs)/SFAs. Comparisons are performed with linear mix models, adjusted by age, sex, and center. *p*-values indicating significant differences are highlighted in bold. Descriptive statistics are shown as mean (SD).

**Table 3:** Dietary intake estimates from the Food Frequency Questionnaire according to the recruiting center.

	London *n* = 47	Barcelona *n* = 50	Paris *n* = 88	Cohen’s f
**BMI**	27.1 (5.1)	27.1 (6.3)	25.7 (6.8)	0.11
**Total energy (kcal/d)**	2265 (746)	2248 (638)	2868 (1004)	**0.36**
**Nutrients**				
Total carbohydrates (% TEI)	53.7 (7.75)	40.5 (6.83)	50.4 (5.91)	**0.77**
Protein (g/weight)	1.34 (0.48)	1.97 (0.80)	2.12 (0.96)	**0.40**
Total fat (% TEI)	35.0 (7.25)	39.6 (6.55)	30.7 (5.56)	**0.60**
SFA (% TEI)	13.5 (3.55)	13.4 (4.42)	14.2 (3.74)	0.09
MUFA (% TEI)	11.7 (2.78)	20.4 (5.66)	9.70 (1.83)	**1.31**
PUFA (% TEI)	4.31 (1.08)	7.48 (3.09)	3.94 (1.11)	**0.82**
Cholesterol (mg/d)	211 (100)	448 (161)	364 (182)	**0.55**
Fiber (g/d)	29.9 (13.6)	25.8 (12.4)	39.2 (19.7)	**0.36**
Sugar (g/d)	116 (63.4)	107 (51.6)	150 (61.2)	**0.32**
**Micronutrients**				
Folic acid (µg/d)	369 (157)	454 (208)	653 (308)	**0.50**
B12 (µg/d)	3.15 (1.45)	1.38 (0.77)	5.54 (2.36)	**0.97**
Sodium (mg/d)	1670 (597)	2942 (996)	2960 (1379)	**0.50**
Potassium (mg/d)	4279 (1502)	4549 (1641)	4864 (1842)	0.14
Calcium (mg/d)	1241 (496)	1158 (609)	1375 (647)	0.16
Magnesium (mg/d)	374 (149)	441 (181)	436 (156)	0.17
Iron (mg/d)	11.5 (7.84)	15.8 (5.69)	14.4 (5.72)	**0.26**
**Food group composition (Standardized per 1000 kcal)**				
Fruit	128 (105)	179 (102)	169 (103)	0.20
Whole fruit (excluding juices)	88.9 (84.8)	159 (97.2)	124 (85.7)	**0.29**
Total vegetables	182 (100)	246 (110)	169 (111)	**0.30**
Greens and beans	64.4 (38.4)	103 (50.5)	52.3 (44.5)	**0.48**
Whole grains	39.4 (36.7)	17.7 (31.7)	16.4 (22.8)	**0.34**
Dairy	116 (93.4)	171 (137)	185 (108)	0.25
Total protein foods	86.3 (32.9)	163 (48.9)	89.1 (36.2)	**0.86**
Seafood and plant protein	15.0 (15.2)	43.7 (23.3)	37.3 (26.3)	**0.48**
Fatty acids profile *	1.23 (0.26)	2.15 (0.49)	1.01 (0.24)	**1.47**
Refined grains	42.2 (25.6)	49.5 (33.3)	94.0 (42.0)	**0.67**
**Diet Quality**				
Healthy Eating Index 2020	59.0 (8.37)	70.2 (9.88)	54.2 (8.60)	**0.75**

D: Day; TEI: Total Energy Intake; SFA: Saturated Fatty Acids; MUFA: Monounsaturated Fatty Acids; PUFA: Polyunsaturated Fatty Acids * Fatty acids profile: (PUFAs + MUFAs)/SFAs. Values of Cohen’s f larger than 0.25 are highlighted in bold. Descriptive statistics are shown as mean (SD).

## Data Availability

Data will be found at the Zenodo repository for the GO-DS21 project https://zenodo.org/communities/eu/records?q=GO-DS21&l=list&p=1&s=10&sort=bestmatch. This page was created on 22 July 2021.

## Supplementary Materials

The following supporting information can be downloaded at: https://www.mdpi.com/article/10.3390/nu18050779/s1, Figure S1: Distribution of participants according to age; Figure S2: Total protein intake according to the body weight category; Figure S3: Macronutrients intake according to the recruiting country; Table S1: Main comorbidities that may affect the dietary profile of the participants; Table S2: Sex differences in dietary intake estimates from the Food Frequency Questionnaires; Table S3: Summary of the association of IQ with the studied variables.

## Abbreviations

The following abbreviations are used in this manuscript:

DS	Down Syndrome
Go-DS21	Gene Overdosage and Comorbidities During the Early Lifetime in Down syndrome
BMI	Body Mass Index
KCL	King’s College London
HMRI	Hospital del Mar Research Institute
IJL	Institut Jérôme Lejeune
FFQ	Food Frequency Questionnaire
HEI-2020	Healthy Eating Index 2020
PA	Physical activity
VREM	Spanish short version of the Minnesota Leisure Time Physical Activity Questionnaire
METs	Metabolic Equivalent of the Task
EE	Energy Expenditure
IQ	Intellectual Quotient
SD	Standard Deviation
PUFAs	Polyunsaturated Fatty Acids
MUFAs	Monounsaturated Fatty Acids
SFAs	Saturated Fatty Acids
