# Supplementary material for: Dietary Patterns and Lifestyle Factors as Determinants of Body Mass Index and Body Composition in Individuals with Down Syndrome—A Study Across Three Clinical Sites
Source: Nutrients. 2026 Feb 27;18(5):779. doi: 10.3390/nu18050779 (PMC12987271; doi:10.3390/nu18050779)
Supplement: Supplementary file 1 [file nutrients-18-00779-s001.zip › nutrients-4135260-supplementary.pdf]

Supporting information

GO-DS21 Consortium:

Yann Herault
Université de Strasbourg, CNRS, INSERM, Institut de Génétique et de Biologie Moléculaire et Cellulaire (IGBMC), Department of Translational Medicine and Neurogenetics, 1 rue Laurent Fries, 67404 IllkirchGraffenstaden, France.
Université de Strasbourg, CNRS, INSERM, CELPHEDIA, PHENOMIN-Institut Clinique de la Souris (ICS), 1 rue Laurent Fries, 67404 Illkirch-Graffenstaden, France.

Andre Strydom
Institute of Psychiatry, Psychology, and Neuroscience, King's College London, London, UK.
South London and Maudsley NHS Foundation Trust, London, UK.
The LonDowns Consortium, London, UK.

Li Chan
Centre for Endocrinology, William Harvey Research Institute, Barts and the London School of Medicine, Queen Mary, University of London, Charterhouse Square, London, UK.

Marie-Claude Potier
Paris Brain Institute, ICM, Pitié-Salpêtrière Hospital, Paris, France.

Johannes Beckers
Institute of Experimental Genetics, Helmholtz Zentrum München – German Research Center for Environmental Health, Ingolstaedter Landstr. 1, 85764 Neuherberg, Germany.
German Center for Diabetes Research (DZD), Ingolstaedter Landstr. 1, 85764 Neuherberg, Germany. Chair of Experimental Genetics, School of Life Sciences Weihenstephan, Technische Universität München, Alte Akademie 8, 85354 Freising, Germany.

Pietro Liò
Department of Computer Science and Technology, University of Cambridge, Cambridge, UK.

Mara Dierssen
Centre for Genomic Regulation (CRG), The Barcelona Institute of Science and Technology, Barcelona, Spain.
Universitat Pompeu Fabra (UPF), Barcelona, Spain.
Centro de Investigación Biomédica en Red de Enfermedades Raras (CIBERER), Barcelona, Spain

Supplementary Figure S1

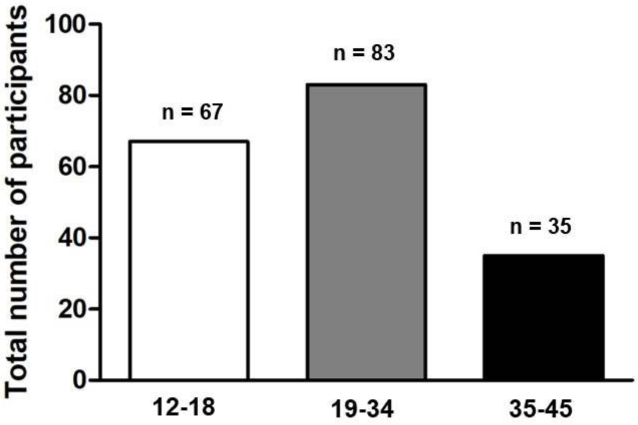

Figure S1. **Distribution of the participants by age**. Participants included in the study were classified considering three age ranges: adolescences (12-18 years old), young adults (19-34 years old) and adults (35-45 years old).

Supplementary Figure S2

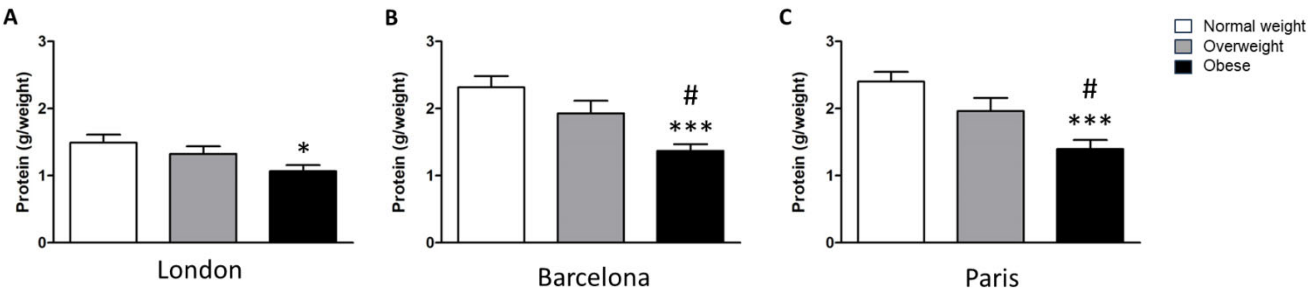

Figure S2. **Total protein intake according to the body weight category.** Total protein consumption presents a significant decreasing trend with increasing BMI, in participants from London (A), Barcelona (B) and Paris (C). Significant differences were considered when $p < 0.05$. * $p < 0.05$, *** $p < 0.001$ (compared to normal weight); # $p < 0.05$ (compared to overweight).

Supplementary Figure S3

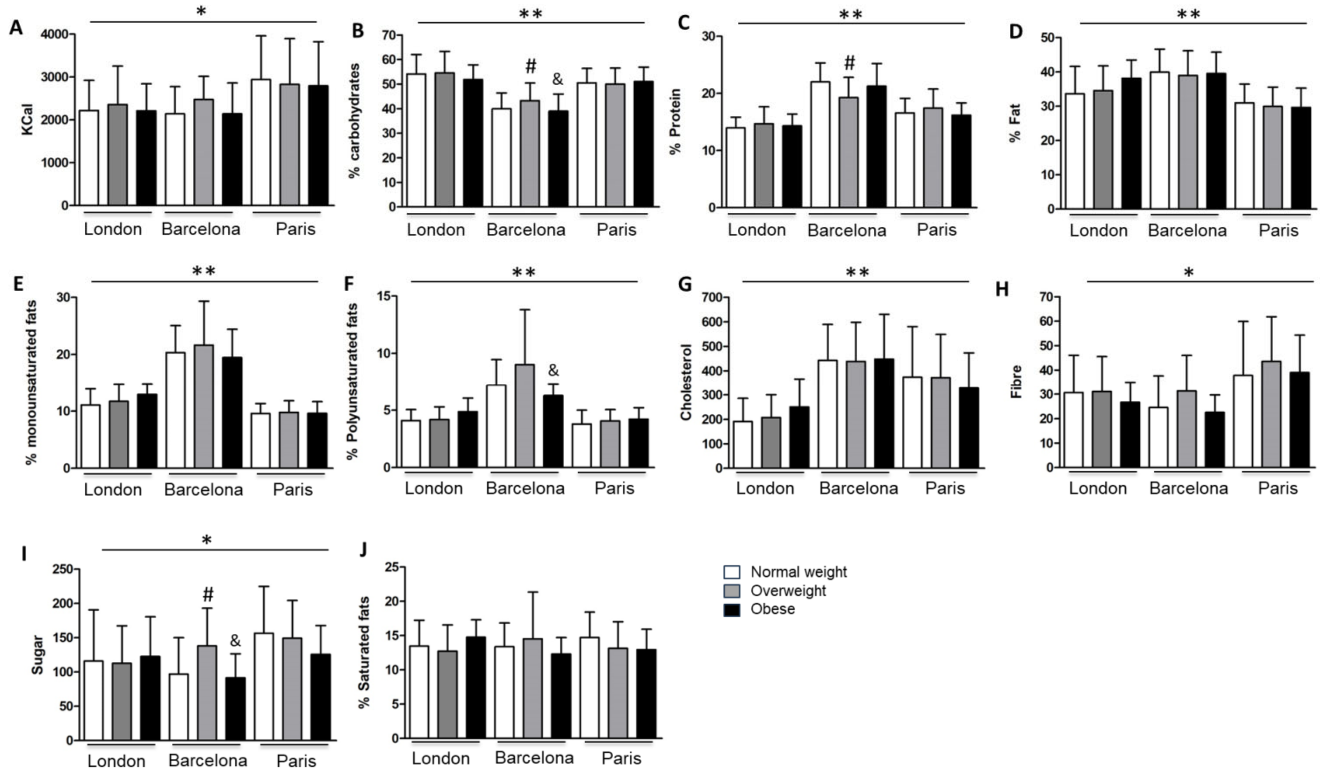

Figure S3. **Macronutrients intake according to the recruiting country**. (A) Large differences between countries were observed in the total caloric intake, as well as in the consumption of different macronutrients including: (B) % of carbohydrates, (C) % of protein, (D) % of fat, (E) % of monounsaturated fat, (F) % of monounsaturated fat, (G) cholesterol, (H) fiber and (I) sugar. (J) No differences were found for saturated fatty acids. Data is presented as mean and standard deviation. Differences were considered when f > 0.25 (* f > 0.25 (medium); ** f > 0.40 (large) (comparisons between countries)), or when p < 0.05 (# p < 0.05 (normal weight vs overweight; & p < 0.05 (overweight vs obese).

Table S1. Main comorbidities that may affect the dietary profile of participants

Comorbidity	Total number of participants
Food allergy	12
Hypertension	2
History of severe lactose intolerance	10
Celiac disease	12
Gastroesophageal reflux	34
Fatty liver disease	2
Chronic constipation	24
Diabetes type 1	1
Diabetes type 2	1
Hypothyroidism	94
Hyperthyroidism	6
Obesity	23
Hyperlipidaemia	6
Depression	18
Anxiety	12
Kidney disease	1

Main comorbidities observed among study participants that may have an impact on their dietary profile. Values represent the total number of participants presenting each condition.

Table S2. Sex differences in dietary intake estimates from the Food Frequency Questionnaires.

	**Female N=83**	**Male N=102**	**P value**
**BMI**	27.4 (6.77)	25.7 (5.83)	**0.009**
**Total energy (kcal/d)**	2406 (885)	2662 (908)	0.052
**Nutrients**			
Total carbohydrates (% TEI)	48.0 (8.95)	49.0 (7.85)	0.494
Protein (g/weight)	1.92 (0.91)	1.85 (0.85)	0.912
Total Fat (% TEI)	34.5 (8.06)	33.9 (6.63)	0.729
SFA (% TEI)	13.6 (3.65)	14.0 (4.07)	0.342
MUFA (% TEI)	13.2 (5.67)	13.0 (5.78)	0.747
PUFA (% TEI)	4.87 (1.82)	5.09 (2.79)	0.271
Cholesterol (mg/d)	334 (159)	359 (197)	0.209
Fibre (g/d)	34.1 (20.9)	32.5 (14.2)	0.311
Sugar (g/d)	119 (57.3)	138 (66.5)	**0.049**
**Micronutrients**			
Folic acid (µg/d)	520 (324)	533 (238)	0.903
B_{12} (µg/d)	3.59 (2.28)	3.99 (2.74)	0.357
Sodium (mg/d)	2493 (1128)	2737 (1339)	0.122
Potassium (mg/d)	4524 (1853)	4717 (1600)	0.575
Calcium (mg/d)	1188 (460)	1359 (695)	**0.050**
Magnesium (mg/d)	421 (179)	422 (149)	0.926
Iron (mg/d)	14.0 (7.2)	14.1 (5.9)	0.923
Fruit	174 (104)	151 (104)	0.098
Whole fruit (excluding juices)	139 (95.8)	113 (87.2)	**0.024**
Total Vegetables	204 (101)	184 (120)	0.142
Greens and beans	72.4 (47.8)	66.6 (50.7)	0.376
Whole grains	28.4 (34.6)	17.8 (26.6)	**0.015**
Dairy	155 (98.6)	171 (130)	0.296
Total Protein Foods	119 (59.5)	99.8 (42.3)	**0.002**
Seafood and plant protein	38.9 (29.5)	28.7 (20.7)	**0.001**
Fatty acids profile *	1.39 (0.59)	1.36 (0.58)	0.730
Refined Grains	66.2 (42.8)	70.7 (43.9)	0.437
**Diet Quality**			
Healthy Eating Index 2020	61.6 (10.4)	58.2 (11.5)	**0.008**

D: Day; TEI: Total Energy Intake; SFA: Saturated Fatty Acids; MUFA: Monounsaturated Fatty Acids; PUFA: Polyunsaturated Fatty Acids *Fatty acids profile: (PUFAs + MUFAs)/SFAs. Comparisons adjusted by age and recruiting centre.

Table S3. Summary of the association of IQ with the studied variables

Variable	Beta coefficient IQ	P-value IQ	R-squared model
BMI	-0.151	0.132	-
Energy (kcal/day)	-4.222	0.688	-
**Nutrients**			
Total Carbohydrates %	0.394	**0.045**	0.137
Protein (g/kg)	0.001	0.916	-
Total fat %	-0.157	0.151	-
SFA	-0.121	0.110	-
Cholesterol (mg)	-3.698	0.116	-
Fibre	0.517	**0.010**	0.236
**Micronutrients**			
Folic acid	6.968	**0.035**	0.242
Vitamin B12	0.011	0.406	-
Sodium	-0.081	0.989	-
**Food group composition**			
Fruit	1.759	0.303	-
Whole fruit	1.381	0.386	-
Total vegetables	4.098	**0.031**	0.106
Greens and beans	1.187	0.180	-
Whole grains	0.544	0.320	-
Dairy	-0.773	0.747	-
Total Protein foods	0.471	0.530	-
Seafood and plant protein	1.012	**0.004**	0.215
Fatty acids	0.017	**0.029**	0.152
Refined Grains	-0.240	0.673	-
Added sugar	0.056	0.474	-
**Diet Quality**			
HEI score	0.300	0.068	0.165
**Anthropometric measurements**			
Height	0.242	**0.031**	0.458
Hip circumference	-0.188	0.375	-
Waist circumference	-0.279	0.169	-
Weight	-0.163	0.478	-
Body fat %	-0.253	0.078	0.386
Visceral fat score	-0.097	0.160	-

Results of linear regression analysis assessing the association between the studied variables and IQ, adjusted for age and sex (n = 48). Beta coefficients represent the standardized change in IQ associated with a one-standard deviation increase in the independent variable. R-squared values are reported for statistically significant or marginally significant associations.
